# Characterization of recombinant gorilla adenovirus HPV therapeutic vaccine PRGN-2009

**DOI:** 10.1172/jci.insight.141912

**Published:** 2021-04-08

**Authors:** Samuel T. Pellom, Claire Smalley Rumfield, Y. Maurice Morillon, Nicholas Roller, Lisa K. Poppe, Douglas E. Brough, Helen Sabzevari, Jeffrey Schlom, Caroline Jochems

**Affiliations:** 1 Laboratory of Tumor Immunology and Biology, Center for Cancer Research, National Cancer Institute (NCI), NIH, Bethesda, Maryland, USA.; 2 Precigen Inc., Germantown, Maryland, USA.

**Keywords:** Immunology, Oncology, Cancer immunotherapy, Cervical cancer, Head and neck cancer

## Abstract

There are approximately 44,000 cases of human papillomavirus–associated (HPV-associated) cancer each year in the United States, most commonly caused by HPV types 16 and 18. Prophylactic vaccines successfully prevent healthy people from acquiring HPV infections via HPV-specific antibodies. In order to treat established HPV-associated malignancies, however, new therapies are necessary. Multiple recombinant gorilla adenovirus HPV vaccine constructs were evaluated in NSG-β2m^–/–^ peripheral blood mononuclear cell–humanized mice bearing SiHa, a human HPV16^+^ cervical tumor, and/or in the syngeneic HPV16^+^ TC-1 model. PRGN-2009 is a therapeutic gorilla adenovirus HPV vaccine containing multiple cytotoxic T cell epitopes of the viral oncoproteins HPV 16/18 E6 and E7, including T cell enhancer agonist epitopes. PRGN-2009 treatment reduced tumor volume and increased CD8^+^ and CD4^+^ T cells in the tumor microenvironment of humanized mice bearing the human cervical tumor SiHa. PRGN-2009 monotherapy in the syngeneic TC-1 model also reduced tumor volumes and weights, generated high levels of HPV16 E6–specific T cells, and increased multifunctional CD8^+^ and CD4^+^ T cells in the tumor microenvironment. These studies provide the first evaluation to our knowledge of a therapeutic gorilla adenovirus HPV vaccine, PRGN-2009, showing promising preclinical antitumor efficacy and induction of HPV-specific T cells, along with the rationale for its evaluation in clinical trials.

## Graphical abstract

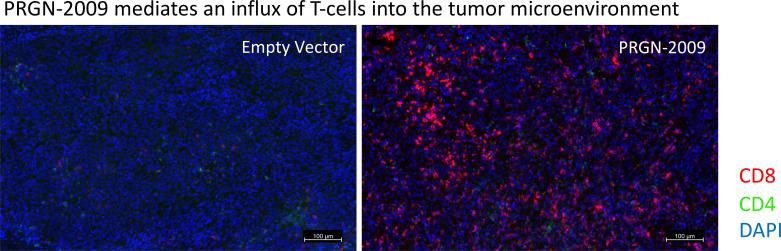

## Introduction

The human papillomavirus (HPV) infects millions of individuals and causes over 600,000 cases of HPV-associated malignancies worldwide each year (1). There are approximately 44,000 new cases of HPV-associated malignancies diagnosed in the United States annually (2). Although many papillomavirus infections are benign and will resolve on their own, some persistent infections will evolve into epithelial cell dysplasia and may result in cancer of the cervix, vulva, penis, oropharyngeal cavity, head and neck, and anal cavity (3).

Over 200 types of HPV are categorized into high-risk and low-risk groups depending on their oncogenic potential (4). Among the high-risk HPV types, HPV types 16 and 18 are the most prevalent and carcinogenic (5, 6). Together, HPV 16 and 18 are responsible for approximately 70% of cervical cancer cases (7). Early (E) HPV genes (E1–E8) regulate viral expression and replication, and late (L) genes control viral protein coding (8–10). Several prophylactic HPV vaccines developed to target the major capsid protein L1 of the HPV viral particle have been shown to be effective and represent a major breakthrough in the reduction of HPV malignancies in developed countries (7). Prophylactic vaccines prevent healthy individuals from acquiring HPV infections, and they prevent previously infected individuals from being reinfected. Unfortunately, prophylactic vaccines are not effective against established HPV infections and the resultant HPV-associated malignant or premalignant lesions (11, 12). Unlike prophylactic HPV vaccines, which are used to generate neutralizing antibodies against the viral capsid protein L1 to prevent infection (13), therapeutic HPV vaccines are used to stimulate cell-mediated immune responses to specifically target and kill infected cells.

The E6 and E7 proteins of HPV 16 and 18 represent potential targets for therapeutic vaccines because they are responsible for maintenance of the malignant phenotype and are constitutively expressed in the tumors (12). Moreover, they are not endogenously expressed on any human tissues, so there is very low risk of inducing autoimmune events with a vaccine targeting these proteins. Several CD8^+^ T cell epitopes of E6 and E7 capable of eliciting cytotoxic T lymphocyte (CTL) responses have previously been identified (14–16), and clinical studies employing diverse vaccine platforms have demonstrated various degrees of effectiveness in terms of eliciting HPV-specific responses and clinical benefits. These include live vector, peptide or protein, cell-based, and nucleic acid vaccines (12, 17–24). The majority of these vaccines target HPV oncoproteins E6 and E7 with the goal to activate HPV antigen–specific CD8^+^ cytotoxic T cells or CD4^+^ helper T cells. These therapeutic vaccines also differ by their routes of administration.

In this study, we evaluated PRGN-2009, which is based on GC46, a gorilla adenovirus vector originally identified and isolated from nonhuman primate sources and vectorized. GC46 has the capability of encoding a large payload (25) and has been shown to have low seroprevalence in the human population. It has been hypothesized that positive titers may be too low to be inhibitory to vaccination (26). GC46 used in these studies is deleted in 2 essential viral regions (E1 and E4) to prevent viral replication in transduced cells (27).

The current study evaluated multiple recombinant gorilla adenovirus vaccine constructs for in vitro HPV-specific T cell activation and in vivo antitumor efficacy in HPV16^+^ mouse models in order to select a construct to move forward into additional preclinical studies. TC-1 is a well-established murine HPV16^+^ lung cancer cell line that is widely used for HPV-associated malignancy research. SiHa is a human HPV16^+^ cervical tumor cell line that is used in the NSG-β2m^–/–^ peripheral blood mononuclear cell (PBMC) humanized mouse model. The humanized mouse model is used to better visualize the patients’ immune responses by utilizing a human tumor in a mouse that has been reconstituted with human immune cells and treating with human specific immuno-oncology agents.

## Results

### Design of the recombinant gorilla adenovirus vaccine PRGN-2009 and antitumor studies in a humanized mouse model.

**Figure 1:**
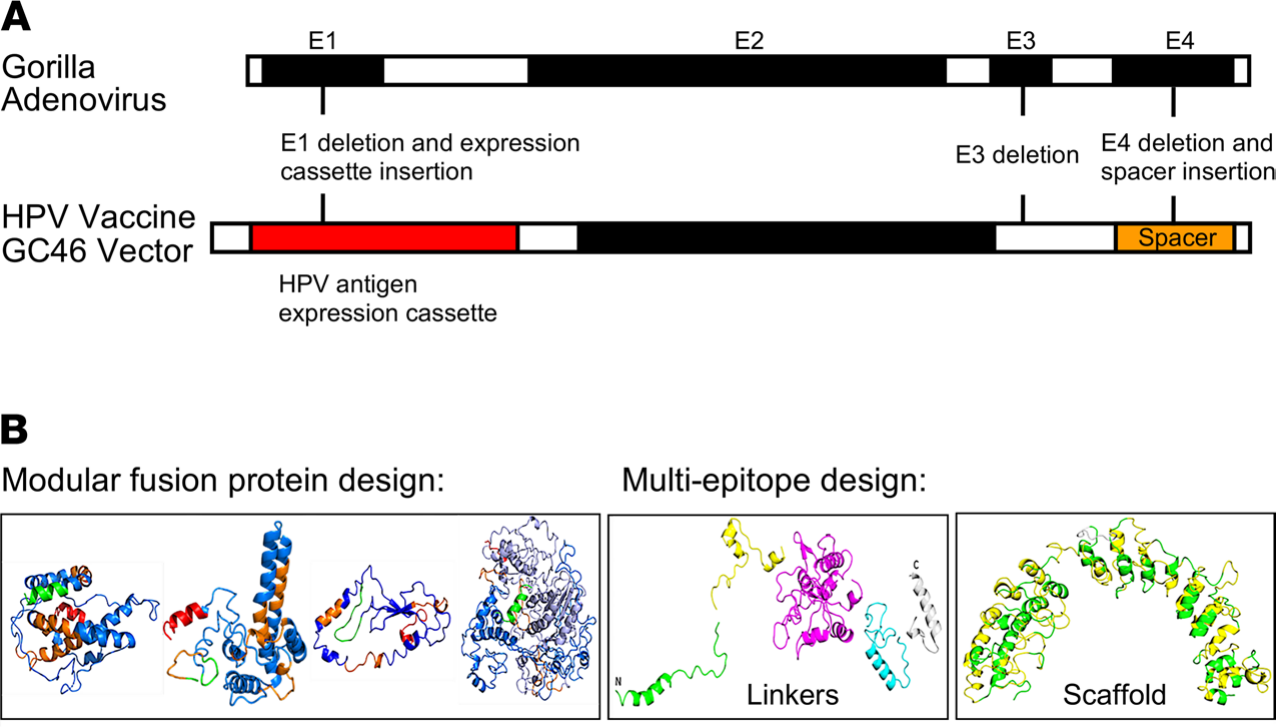
Design of the recombinant gorilla adenovirus vaccine PRGN-2009. (**A**) Schematic representation of the multigene-deleted gorilla adenovector GC46. (**B**) Schematic structural representations of the HPV antigen constructs.

PRGN-2009 is constructed on Precigen Inc.’s nonhuman primate adenoviral vector platform GC46 (25, 26). This platform was engineered to have deletions of 2 regions of the adenovector, E1 and E4, which are essential elements necessary for viral replication; thus, PRGN-2009 is not replication competent (Figure 1A). Initially, 5 different HPV antigen transgene designs (nos. 1–5) were developed and optimized using bioinformatics and Precigen’s UltraVector technology to incorporate into the multigenic large capacity gorilla adenovector. Two constructs (nos. 1 and 3) are modular HPV16 and HPV18 fusion protein designs. Three constructs (nos. 2, 4, and 5) are multi-CTL epitopes grafted onto scaffolds or connected by linkers (Figure 1B). Initial experiments were performed to choose the HPV antigen design that demonstrated the best in vitro T cell activation specific to HPV and the best antitumor efficacy potential to move forward with all subsequent experimentation and analyses.

**Figure 2:**
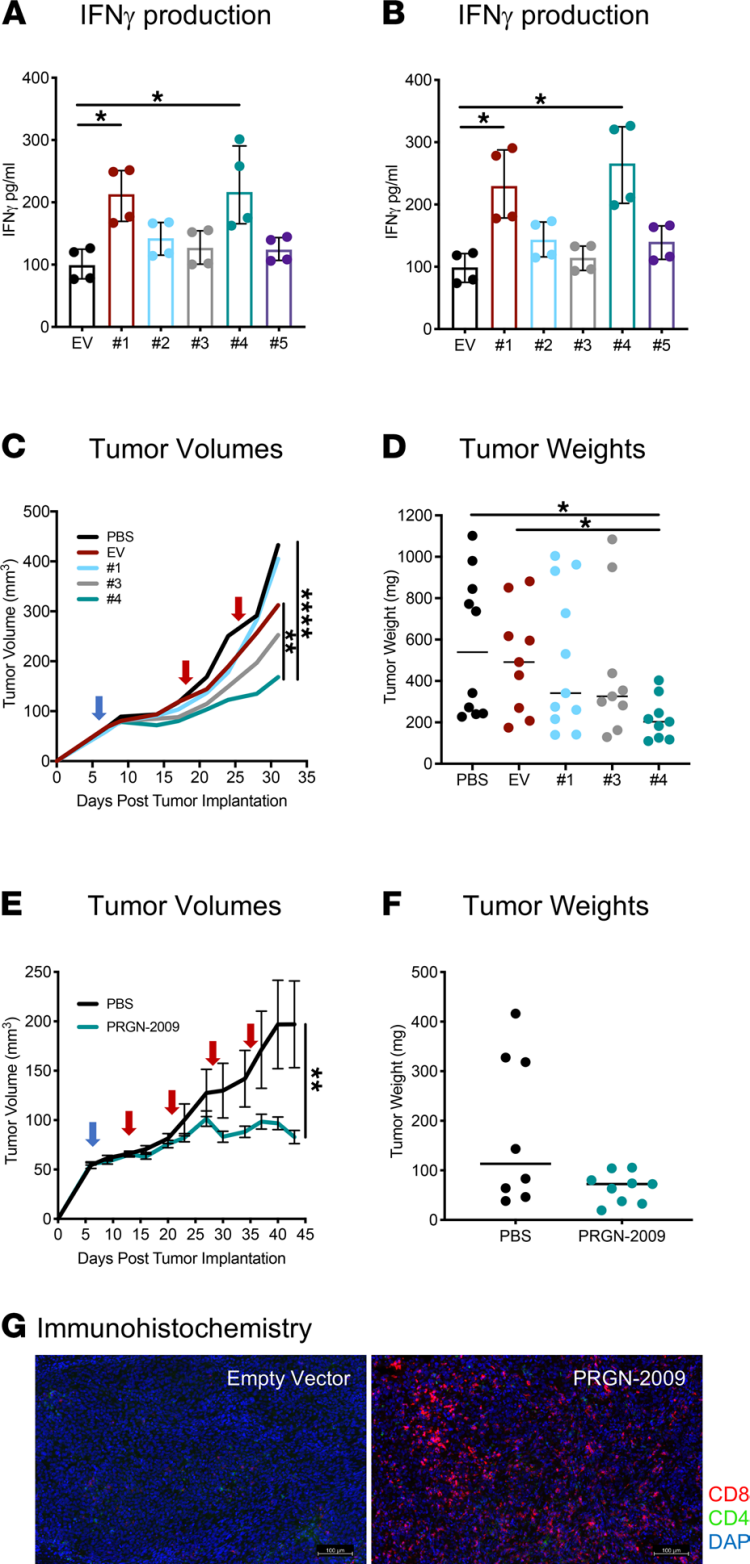
Antitumor studies of PRGN-2009 in a humanized mouse model. (**A** and **B**) HPV-specific IFN-γ production from healthy-donor PBMCs stimulated by autologous DCs infected with HPV vaccine constructs, nos. 1–5, for 2 IVS cycles and restimulated twice with overlapping HPV16 E6/E7 15-mer peptides. IFN-γ levels in supernatants 24 and 48 hours after the second restimulation are shown for HPV16 E6 (**A**) and HPV16 E7 (**B**). (**C** and **D**) NSG-β2m^–/–^ mice bearing HPV^+^ cervical cancer (SiHa) were reconstituted with human PBMCs (day 7, blue arrow) and treated twice with weekly PBS (100 μL), empty vector (1 × 10^9^ VP), or HPV vaccine constructs (nos. 1, 3, and 4; 1 × 10^9^ VP) s.c. (red arrows). (**C**) Tumor volumes. (**D**) Tumor weights on day 31. (**E** and **F**) Humanized NSG-β2m^–/–^ mice bearing SiHa tumors were vaccinated 4 times with PRGN-2009 (construct no. 4). (**E**) Tumor volumes. (**F**) Tumor weights on day 43. (**G**) Tumors from **C** were dissected and stained using the Perkin Elmer Opal IHC kit. Central tumor sections are shown for a representative mouse from the empty vector and PRGN-2009 groups. Red, CD8^+^ T cells; green, CD4^+^ T cells; blue, DAPI. Medians are shown. Kruskal-Wallis and repeated measures ANOVA. *P < 0.05, **P < 0.01, ****P < 0.0001. EV, empty vector; IVS, in vitro stimulation; PBMCs, peripheral blood mononuclear cells; PBS, phosphate buffered saline; VP, virus particles. Scale bars: 100 μm.

Healthy-donor PBMCs were stimulated in vitro by autologous DCs infected with the 5 different HPV vaccine constructs (nos. 1–5) as described in Methods. Following these stimulations, an IFN-γ production ELISA assay using overlapping HPV16 E6 and E7 15-mer peptides was performed to detect peptide-specific T cell stimulation. The IFN-γ levels in the supernatant of PBMCs stimulated with 2 of the HPV vaccine constructs (nos. 1 and 4) were increased compared with the empty vector control and other constructs when restimulated with the HPV16 E6 15-mer peptides (Figure 2A, *P <* 0.05) and the HPV16 E7 15-mer peptides (Figure 2B, *P <* 0.05).

To determine which HPV vaccine construct demonstrated the best antitumor efficacy in vivo, NSG-β2m^–/–^ mice bearing HPV^+^ cervical cancer (SiHa) were reconstituted with human HLA-A24^+^ PBMCs from a healthy donor on day 7 (Figure 2C, blue arrow) and treated twice with weekly injections of phosphate buffered saline (PBS) control (100 μL), empty vector (1 × 10^9^ vector particles [VP]), or 3 HPV vaccine constructs (nos. 1, 3, or 4) (1 × 10^9^ VP) given s.c., as indicated by red arrows (Figure 2C). Based on the tumor volumes for the duration of the study, mice treated with the PRGN-2009 HPV vaccine construct (no. 4) displayed the lowest tumor growth rate compared with all other treatment groups (Figure 2C). The tumor volumes of mice treated with PRGN-2009 (no. 4) were significantly lower than PBS control–treated (*P <* 0.0001) and empty vector control–treated (*P <* 0.01) mice. At the end of study, mice treated with PRGN-2009 (no. 4) also displayed the lowest tumor weights (*P <* 0.05 versus PBS and empty vector controls, Figure 2D). An additional experiment was performed in the same human cervical cancer model — this time, vaccinating mice 4 times on a weekly basis with PRGN-2009 (no. 4). NSG-β2m^–/–^ mice treated with PRGN-2009 (no. 4) displayed significantly lower tumor volumes (*P <* 0.01) compared with PBS control–treated mice (Figure 2E). There was a clear trend of decreased tumor weights at the end of study with PRGN-2009 treatment; however, there were no significant differences between treatment groups in this experiment (Figure 2F). Tumors from these humanized mice were dissected at day 31 and stained by IHC. Sections from the center of the tumors showed that s.c. HPV vaccine treatment greatly upregulated CD8^+^ T cells in the tumor microenvironment (TME) compared with empty vector–treated mice (Figure 2G).

The above data suggest that the PRGN-2009 HPV vaccine construct (no. 4) was the most efficacious both in vitro by IFN-γ ELISA and in vivo by decreased mouse tumor volumes and tumor weights at the end of the study, and it promoted increased CD8^+^ T cell infiltration. HPV vaccine construct PRGN-2009 (no. 4) was therefore used in all subsequent experiments and is referred to as PRGN-2009. The higher immune and therapeutic responses seen with the PRGN-2009 vaccine construct in these studies is likely due to the molecular design of the antigen and the position of the antigen components according to Precigen’s bioinformatics analyses, which created optimum presentation. The PRGN-2009 antigen design contains 35 non–HLA-restricted CTL epitopes of HPV 16 and 18, including 3 HLA-A2 agonist epitopes of HPV16 previously identified (28), constitutively expressed under control of a cytomegalovirus (CMV) promotor, and linked based on Precigen’s bioinformatics process. These peptide sequences are linked to human ankyrin repeat protein scaffold, enabling protein linker sequences embedded between the peptides, whereby this scaffold retains its tertiary structure displaying the HPV epitopes (Figure 1B, right). Designed shuffling of the peptides prevents any reformation of oncogenic protein potential and HPV protein viral function.

### Treatment with the recombinant gorilla adenovirus vaccine PRGN-2009 resulted in decreased tumor volumes and induction of antigen-specific T cells in the TC-1 syngeneic mouse model.

**Figure 3:**
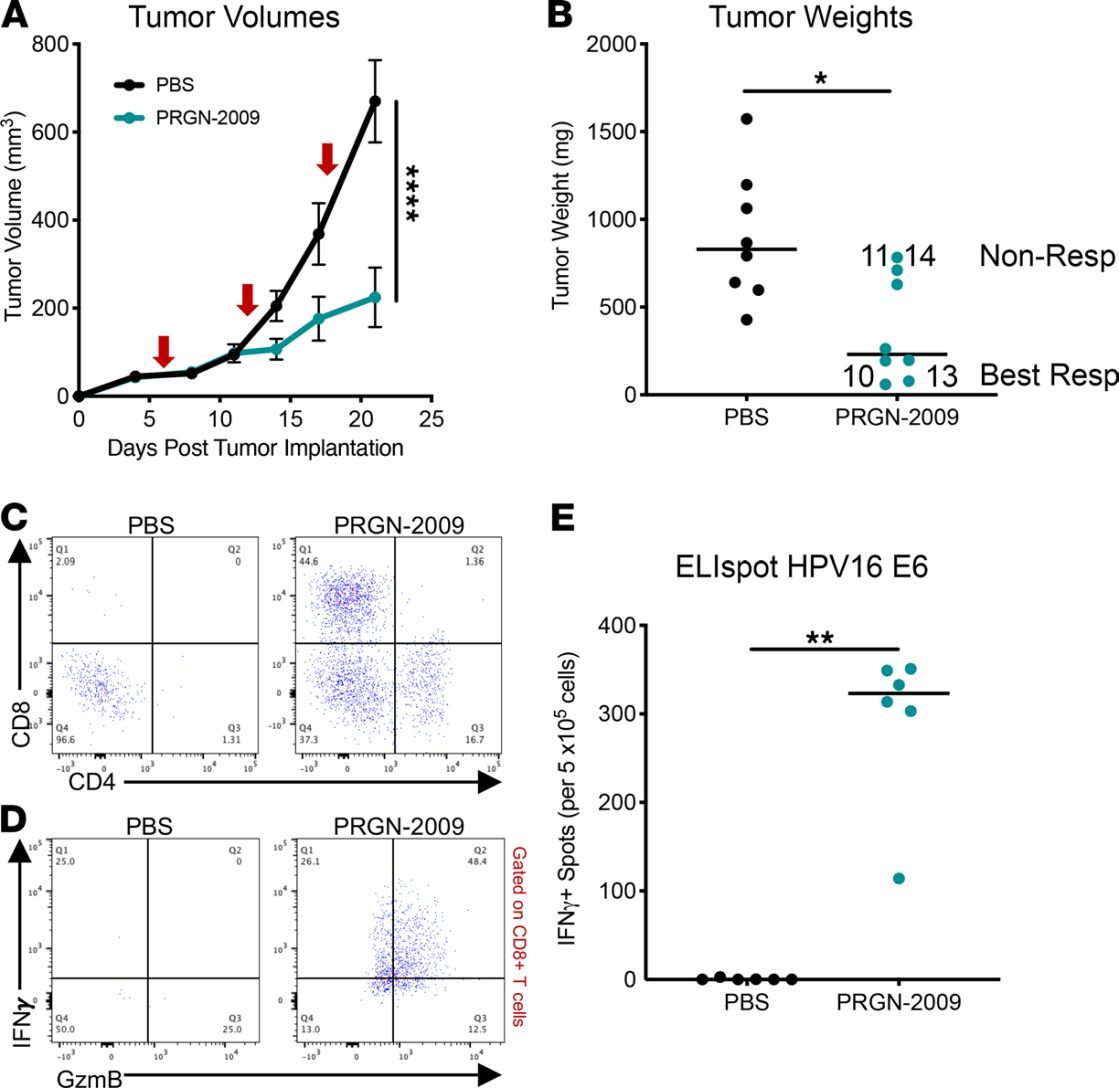
Treatment with PRGN-2009 resulted in decreased tumor volumes and induction of antigen-specific T cells in the TC-1 syngeneic mouse model. C57BL/6 mice bearing s.c. TC-1 HPV16^+^ tumors were treated with 3 weekly s.c. injections of PBS control (100 μL) or PRGN-2009 (1 × 10^9^ VP), starting on day 4 after tumor implantation (red arrows). (**A**) Average tumor volumes of PRGN-2009 versus control-treated mice; *P <* 0.0001. (**B**) Tumor weights (medians) at the end of study; *P <* 0.05. Mice nos. 10 and 13 were defined as Best Responders, with tumor weights below the group median, and mice nos. 11 and 14 were defined as Nonresponders, with tumor weights above the group median (Table 1). (**C** and **D**) Flow cytometry of single-cell suspensions of tumors. (**C**) CD8^+^ and CD4^+^ T cell tumor infiltration are shown from a representative mouse from each group. (**D**) Multifunctional (IFN-γ^+^GzmB^+^) CD8^+^ T cell infiltration from 1 representative mouse from each group. (**E**) Splenocytes from 6 mice from each group were stimulated with overlapping HPV16 E6 15-mer peptides in an ELIspot assay. The graph shows the number of SFCs per 2.5 × 10^5^ splenocytes of PRGN-2009– versus control-treated mice after deducting the negative control. Mann-Whitney *U* and repeated measures ANOVA tests were used. *P < 0.05, **P < 0.01, ****P < 0.0001. SFCs, spot-forming cells; VP, virus particles.

The syngeneic mouse model consisting of HPV^+^ TC-1 tumors has been used extensively to study various HPV vaccines and was used in further studies. C57BL/6 mice bearing s.c. TC-1 HPV16^+^ tumors were treated with 3 weekly injections of PBS control or PRGN-2009 (1 × 10^9^ VP, s.c.). After the second injection, PRGN-2009–treated mice had significantly lower tumor volumes compared with PBS control–treated mice (*P <* 0.0001, Figure 3A) and also displayed significantly lower tumor weights at the end of study compared with PBS control–treated mice (*P <* 0.05, Figure 3B).

To evaluate T cell infiltration into the TME, flow cytometry of single-cell suspensions of tumors was performed. Following PRGN-2009 treatment, there was an increase in tumors in both CD8^+^ T cell infiltration (44.6% of all live cells versus 2.09% in control treated mice) and CD4^+^ T cell infiltration (16.7% versus 1.31% of all live cells) (Figure 3C). The infiltration of multifunctional (IFN-γ^+^GzmB^+^) CD8^+^ T cells, which have previously been shown to be cytolytic (29), into tumor was also greatly increased following PRGN-2009 treatment (48.4%) compared with PBS control (0%) (Figure 3D). To evaluate T cell antigen specificity generated by PRGN-2009 treatment, splenocytes were isolated from mice from both treatment groups for analysis using IFN-γ ELIspot. Overlapping 15-mer peptides from the HPV16 E6 protein were used as the target antigen. Only mice treated with PRGN-2009 developed significant antigen-specific responses against the HPV16 E6 peptides (*P <* 0.01) (Figure 3E).

Table 1 shows the numbers of several immune cell subsets in the tumor from this experiment. When comparing all PRGN-2009–treated mice (*n* = 8) to PBS-treated controls (*n* = 8, Table 1, top), total CD8^+^ T cells per mg of tumor increased greatly, to a 33:1 ratio. This was also seen for total CD4^+^ T cells per mg of tumor, which increased to a ratio of 13:1. As has previously been observed in multiple clinical trials (30, 31), we found that the number of Tregs increased when the total CD4^+^ T cell population increased. Furthermore, the multifunctional (IFN-γ^+^GzmB^+^) CD8^+^ T cells were detected at 40-fold higher levels in PRGN-2009–treated mice, and the activated (PD-1^+^) CD8^+^ T cells were seen at 70-fold higher levels; these 2 subsets were below the detection level in control mice. It should be noted (Figure 3B) that there was a clear distinction in 2 groups of mice in antitumor effects after vaccination. Mice were thus designated as “Best Responders” or “Nonresponders” depending on their tumor weights at the end of study in an attempt to define whether differences exist between these 2 groups in terms of tumor immune infiltrate. Best Responder mice (nos. 10 and 13, Figure 3B) displayed tumor weights below the PRGN-2009 group median, and Nonresponder mice (nos. 11 and 14) had tumor weights above the group median. The multifunctional (IFN-γ^+^GzmB^+^) and activated (PD-1^+^) CD8^+^ T cells increased the most when comparing Best Responder mice to Nonresponders after PRGN-2009 treatment (Table 1, middle). Multifunctional CD8^+^ T cells were detected at 7-fold higher levels in Best Responders, and activated CD8^+^ T cells were detected at 6-fold higher levels. Total CD8^+^ T cells increased to a 5:1 ratio. When comparing PRGN-2009–treated mice (Best Responders only) with PBS controls (Table 1, bottom), the previously stated trends continued to expand, with approximately 50 times more CD8^+^ T cells per mg of tumor in Best Responder mice compared with controls, and even higher ratios for multifunctional and activated CD8^+^ T cells (Table 1, bottom). The single-positive CD8^+^IFN-γ^+^ T cells were increased in Best Responder PRGN-2009–treated mice compared with Nonresponder PRGN-2009–treated mice to a ratio of 8:1, and CD4^+^IFN-γ^+^ T cells were increased to a ratio of 5:1 (Supplemental Table 1, middle; supplemental material available online with this article; https://doi.org/10.1172/jci.insight.141912DS1). When comparing these immune cell subsets between all PRGN-2009–treated mice (*n* = 8) and PBS controls (*n* = 8, Supplemental Table 1, top), they were undetectable in controls but were detected at low levels in vaccinated mice. Additional studies comparing empty vector control to PRGN-2009 treatment showed similar results when comparing the Best Responder and Nonresponder mice, with the highest increases observed for the activated (PD-1^+^) and multifunctional (IFN-γ^+^GzmB^+^) CD8^+^ T cells.

There was a trend toward lower numbers of myeloid-derived suppressor cells (MDSCs) (CD11b^+^Gr1^+^) in mice responding to PRGN-2009 treatment (Supplemental Table 1, middle). In contrast, the MDSC population trended higher for the entire PRGN-2009–treated group compared with control-treated mice (Supplemental Table 1, top). In an additional experiment performed using complementary MDSC markers for granulocytic (CD11b^+^Ly6G^+^) and monocytic MDSCs (CD11b^+^Ly6C^+^), there was a trend of increase in both subsets following vaccine treatment (Supplemental Figure 1, A and B). In the same study, markers specific for M1 and M2 macrophages were used (CD11b^+^F4/80^+^MHC-II^+^ and CD11b^+^F4/80^+^CD206^+^, respectively). As seen in Supplemental Figure 1C, the number of M1 tumor-associated macrophages (TAM) trended higher in PRGN-2009–treated mice, and the number of M2 TAM trended lower after PRGN-2009 treatment (Supplemental Figure 1D). There was a trend toward an increased M1/M2 ratio after treatment with PRGN-2009 (Supplemental Figure 1E, *P* = 0.053). A decreased M1/M2 ratio has previously been shown to correlate with reduced survival and a poor response to chemoradiation in patients with advanced cervical cancer (32). Thus, the increased M1/M2 ratio observed after PRGN-2009 vaccination may indicate a more beneficial TME.

**Figure 4:**
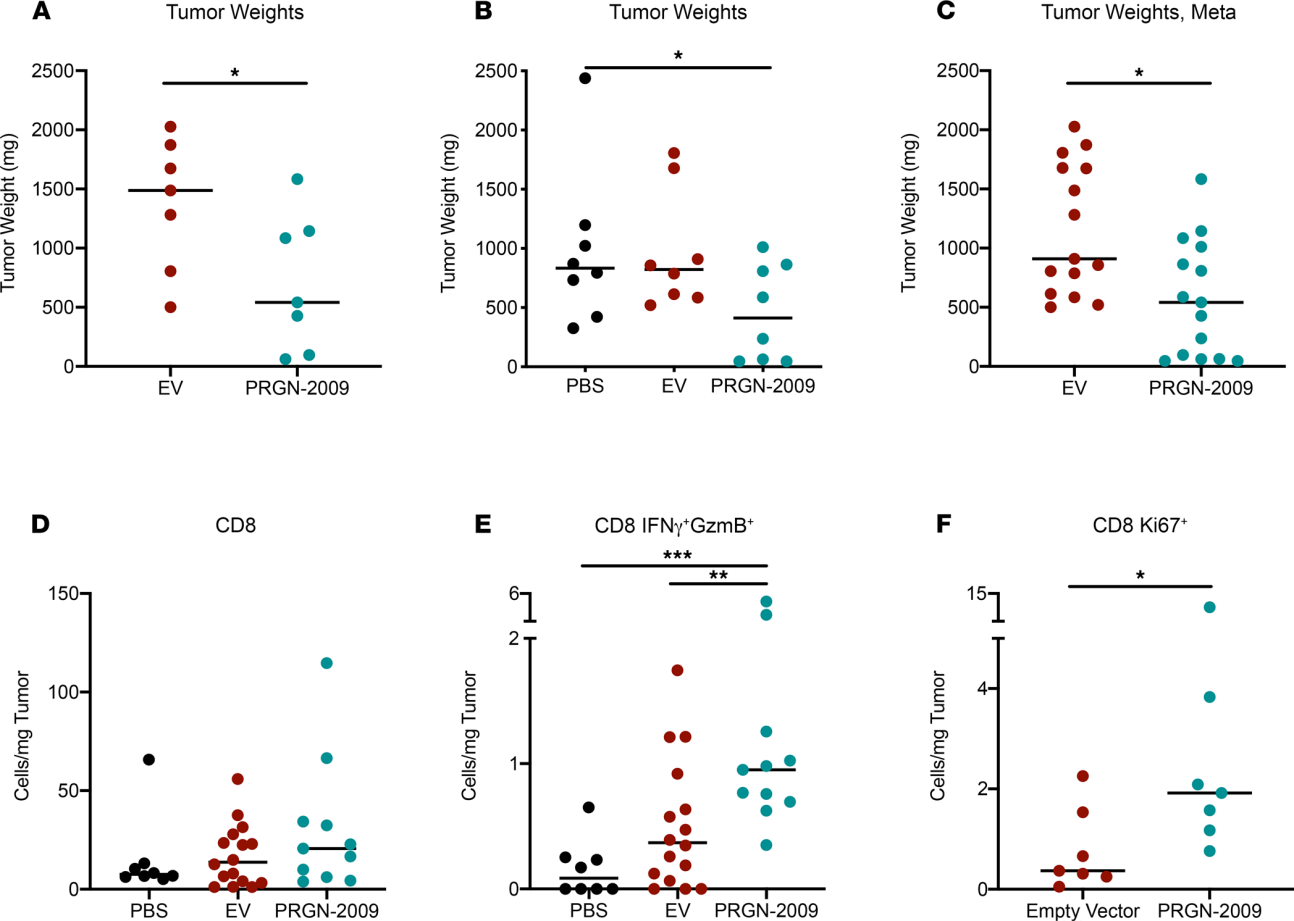
PRGN-2009 treatment resulted in decreased tumor volumes and increased intratumoral immune cell effector subsets. C57BL/6 mice (*n* = 7–8 per group) bearing s.c. TC-1 HPV16^+^ murine tumors were treated in 2 separate studies with empty vector control (1 × 10^9^ VP, s.c.), PBS (100 μL, s.c.), or PRGN-2009 (1 × 10^9^ VP, s.c.) on days 7 and 14 after tumor implantation. (**A**) Study 1, end-of-study tumor weights for individual mice. (**B**) Study 2, end-of-study tumor weights for individual mice. (**C**) Meta-analysis of both studies, showing end-of-study tumor weights. (**D**–**F**) Flow cytometry of single-cell suspensions of tumor tissue. T cell subsets are shown per mg tumor. The Ki67 stain was only performed in 1 study. Figures show the medians. Mann-Whitney *U* and Kruskal-Wallis tests were used. *P < 0.05, **P < 0.01, ***P < 0.001. VP, virus particles.

Additional experiments were performed using the empty GC46 vector as a negative control in C57BL/6 mice bearing s.c. TC-1 HPV16^+^ tumors. Mice were vaccinated with 2 injections of empty vector control or PRGN-2009, and PRGN-2009–treated mice displayed significantly lower tumor weights at the end of study compared with empty vector control (*P <* 0.05, Figure 4, A–C). At the end of the studies, flow cytometry of single-cell suspensions of tumor tissue was performed, and CD8^+^ and CD4^+^ T cell subsets were evaluated. There were no changes in total CD8^+^ T cells in the tumor (Figure 4D), but there were significant increases in multifunctional CD8^+^ T cells (CD8^+^IFN-γ^+^GzmB^+^, Figure 4E) and CD8^+^ T cells with a proliferative capacity (CD8^+^Ki67^+^, Figure 4F) following PRGN-2009 treatment compared with empty vector control. Certain CD4^+^ T cell subsets showed moderate increases after PRGN-2009 treatment, although not to the same extent as the CD8 subsets.

**Figure 5:**
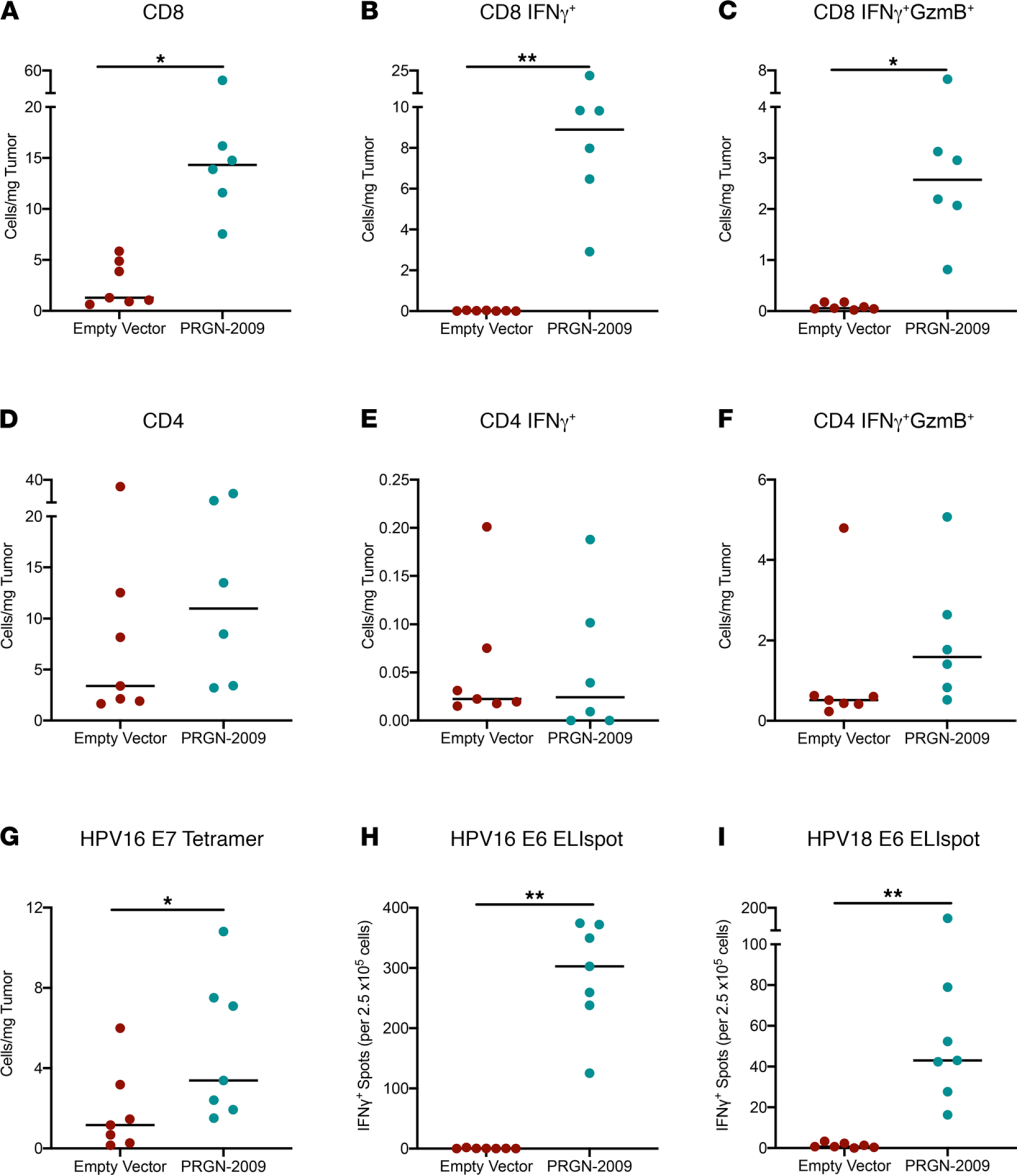
HPV-specific T cells in the TME and periphery induced by PRGN-2009. C57BL/6 mice (*n* = 6–7 per group) bearing s.c. TC-1 HPV16^+^ tumors were treated with empty vector control (1 × 10^9^ VP, s.c.) or PRGN-2009 (1 × 10^9^ VP, s.c.) on days 7 and 14 after tumor implantation. At the end of study (day 23), tumors were harvested. CD45^+^ TILs were isolated and stimulated overnight in triplicate wells with combined overlapping HPV16 E6/E7 15-mer peptides or DMSO (negative control), and they were then evaluated by flow cytometry. (**A**–**F**) Graphs show the number of cells/mg tumor for total CD8 (**A**), IFN-γ–producing CD8 (**B**), IFN-γ plus GzmB–producing CD8 (**C**), total CD4 (**D**), IFN-γ–producing CD4 (**E**), and IFN-γ plus GzmB–producing CD4 (**F**). (**G**) Unstimulated TILs were also evaluated for CD8 and HPV16 E7 tetramer. (**H** and **I**) An ELIspot assay was performed on splenocytes using overlapping 15-mer peptides for HPV16 E6 (**H**) and HPV18 E6 (**I**). Medians are shown. Mann-Whitney *U* test was used. *P < 0.05, **P < 0.01. TILs, tumor-infiltrating lymphocytes; TME, tumor microenvironment; VP, virus particles.

Antigen-specific T cell responses were evaluated both in the tumor and in splenocytes. To evaluate antigen-specific responses in the TME of mice treated with PRGN-2009, CD45^+^ tumor-infiltrating lymphocytes (TILs) were isolated and stimulated overnight in vitro with a mix of HPV16 E6/E7 15-mer peptides. There were not enough TILs available to assay additional HPV antigens. Flow cytometry analysis was performed and showed that PRGN-2009 treatment significantly increased the amount of total CD8^+^ T cells (*P* < 0.05), IFN-γ–producing CD8^+^ T cells (*P* < 0.01), and IFN-γ^+^GranzymeB^+^ (IFN-γ^+^GzmB^+^) multifunctional CD8^+^ T cells (*P* < 0.05) in the TME compared with empty vector treatment (Figure 5, A–C). Similar results were seen with CD4^+^ T cells, but to a lesser degree (Figure 5, D–F). Antigen-specific T cell responses in the TME were further evaluated using a commercially available mouse HPV16 E7 tetramer (RAHYNIVTF). Double staining of TILs showed a significant increase (*P* < 0.05) in CD8^+^tetramer^+^ T cells in the TME of PRGN-2009–treated mice compared with empty vector–treated controls (Figure 5G).

As a measure of peripheral immune responses, splenocytes were isolated from empty vector control and PRGN-2009–treated mice for analysis using IFN-γ ELIspot. Overlapping 15-mer peptides from the HPV 16 and 18 E6/E7 proteins were used as target antigens. PRGN-2009–treated mice developed significant antigen-specific responses against the HPV16 E6 peptides (*P <* 0.01, Figure 5H) and the HPV18 E6 peptides (*P <* 0.01, Figure 5I). Some variations were observed within the groups, which is expected since the results are based on the stimulation of 2.5 × 10^5^ splenocytes per well. Little reactivity to the HPV 16 and 18 E7 peptides was seen using the ELIspot assay (Supplemental Figure 2), but it is important to remember that this is a murine model, and differential epitopes will be recognized in humans. Only PRGN-2009–treated mice displayed IFN-γ production after stimulation. Altogether, tumor growth control and the development of HPV antigen–specific responses induced by PRGN-2009 were reproducible in multiple independent experiments using the TC-1 HPV16^+^ syngeneic mouse model.

### T cell depletion studies and toxicology.

To evaluate the contribution of CD4^+^ and CD8^+^ T cells to the observed antitumor effects of vaccination with PRGN-2009, depletion studies were performed. C57BL/6 mice were depleted of the CD4^+^ or CD8^+^ T cell populations using commercially obtained depleting antibodies prior to instillation of TC-1 tumors. Treatments with PRGN-2009 were started on day 7. At the end of the study, PRGN-2009–treated mice depleted of CD4^+^ T cells displayed larger tumor volumes than nondepleted PRGN-2009–treated mice (Supplemental Figure 3, A and C). Similar trends were also observed in CD8-depleted mice; however, this was to a lesser degree (Supplemental Figure 3, B and C). Thus, both CD4^+^ and CD8^+^ T cells have an impact on the antitumor efficacy of the PRGN-2009 vaccine.

A toxicology study was performed to further evaluate the safety and general tolerability of repeat s.c. administration of PRGN-2009. PRGN-2009 (1 × 10^10^ VP) s.c. was administered once a week for 3 weeks; no significant treatment-related effects in the C57BL/6 mouse were observed based on body weights; full pathological report, including organ weights and histopathology; or full blood and chemical laboratory analyses. Body weights and organ weights from PRGN-2009–treated mice and control mice were in range of one another and not significantly different (Supplemental Table 2). Histopathology analysis was normal between PRGN-2009–treated mice and controls.

## Discussion

Several types of therapeutic HPV vaccines have been evaluated clinically, including peptide, protein, live vector, cell-based, and nucleic acid vaccines (24). The majority of these vaccines target the HPV oncoproteins E6 and E7 with the goal to activate HPV antigen–specific CD8^+^ cytotoxic T cells. HPV E6 and E7 present ideal targets, since they are constitutively expressed on tumor cells but not expressed in healthy tissues.

Peptide- and protein-based vaccines are safe, stable, and easy to produce; to enhance their potency, immunostimulating molecules and adjuvants are added to increase the ability of the vaccines to activate the innate and adaptive immune systems (12, 20). ISA101 is a synthetic long peptide−based vaccine with overlapping peptides to both the HPV16 E6 and E7 proteins, and it is adjuvanted with Montanide emulsifier ISA-51 (Sté Exploitation Produits pour l’Industrie Chimique). ISA101 showed low toxicity and induction of strong T cell responses in a phase I study in women with end-stage cervical cancer (33). Furthermore, ISA101 treatment resulted in complete responses after 1 year in 9 of 19 patients with HPV16^+^ grade 3 vulvar intraepithelial neoplasia (VIN) (34). In contrast, although a phase II study evaluating ISA101 treatment of women with advanced or recurrent HPV16^+^ carcinomas resulted in strong T cell responses, no clinical benefits were seen in this advanced disease setting (35). The efficacy of GL-0810, a therapeutic HPV peptide–based vaccine with adjuvant Montanide and GM-CSF, was evaluated in patients with recurrent/metastatic head and neck squamous cell carcinoma (HNSCC) in a phase I dose-escalation trial (36). Four of the 5 patients who received all 4 vaccinations generated T cell and antibody responses, and no dose-limiting toxicities were observed.

Although viral vector vaccines are highly immunogenic, one challenge is the generation of antivector neutralizing antibodies, which may limit the efficacy of multiple vaccine administrations (24). TG4001, a modified vaccinia Ankara–based (MVA-based) vaccine, contains sequences encoding modified HPV16 E6/E7 and human IL-2. TG4001 was administered s.c. in 21 patients with HPV16-related cervical intraepithelial neoplasia 2/3 (CIN2/3) lesions (37). HPV16 DNA clearance was observed in 8 of 10 responders, HPV16 mRNA clearance in 7, and no recurrence of high-grade lesions was observed for 12 months after treatment.

Nucleic acid–based vaccines are also safe and easy to produce. DNA vaccines are able to sustain antigen expression in cells longer than protein vaccines. A potential limitation is that naked DNA has low intrinsic immunogenicity, since it is unable to amplify and spread from transfected cells to surrounding cells like vector-based vaccines (24). A phase I trial of GX-188E given intramuscularly, followed by electroporation, showed cellular immune responses in 9 patients with CIN3 (38).

The present study is the first to our knowledge to evaluate a recombinant gorilla adenovirus HPV vaccine designated PRGN-2009. Importantly, the HPV antigen design of PRGN-2009 contains 35 CTL epitopes of HPV 16 and 18, including 3 HLA-A2 T cell enhancer agonist epitopes of HPV16 previously identified to augment CTL responses (28). We employed the TC-1 syngeneic tumor model, a murine lung carcinoma transduced with HPV16 E6/E7, often used in HPV^+^ malignancy research. While much information has been gleaned from syngeneic mouse models, they have limitations: they are composed of both murine immune and tumor cells. Moreover, many of the immuno-oncology agents being evaluated are human proteins; therefore, multiple administrations may be hampered by host xenogeneic responses. The NSG-β2m^−/−^ humanized mouse model represents a potential intermediate between syngeneic models and clinical studies. The SiHa human cervical HPV^+^ cancer cell line was employed in humanized NSG mice to investigate human immune cell responses to PRGN-2009.

PRGN-2009 therapeutic vaccination of both syngeneic and humanized mice led to a significant reduction in tumor growth rates compared with PBS control– and empty vector–treated mice. End-of-study tumor weights were also significantly lower in PRGN-2009–treated mice compared with control. Furthermore, PRGN-2009–treated mice had greater numbers of multifunctional (IFN-γ^+^GzmB^+^) and other subsets of CD8^+^ T cells infiltrating the tumor compared with control. In the TC-1 model, only mice treated with PRGN-2009 developed antigen-specific responses against the HPV16 E6 peptides in the TME, measured by CD8/IFN-γ/GzmB staining after in vitro stimulation (IVS) of TILs. Mice treated with PRGN-2009 also displayed HPV16 E7 tetramer^+^ cells in the TME, as well as peripheral T cell responses measured by IFN-γ ELIspot assay of splenocytes stimulated with HPV16 and HPV18 E6 overlapping 15-mer peptides.

Checkpoint inhibitors are now used in frontline therapies against a variety of cancers. Multiple clinical trials are studying checkpoint therapy in the treatment of HPV^+^ malignancies. The results of a trial in 24 patients with HPV16^+^ malignancy, who were treated with the therapeutic HPV vaccine ISA101 in combination with the anti-PD1 checkpoint inhibitor nivolumab, were recently reported (39). Whereas single therapy with either the vaccine or nivolumab yielded minor results, the combination led to a 33% overall response rate (39). This study is important and highlights the potential for combining therapeutic vaccines with checkpoint inhibitors. Bintrafusp alfa is a first-in-class bifunctional fusion protein composed of the extracellular domain of the TGF-βRII to function as a TGF-β “trap” fused to a human IgG1 antibody blocking PD-L1. An ongoing phase I/II clinical trial (NCT02517398; https://clinicaltrials.gov/ct2/show/NCT02517398) in patients with HPV-associated malignancies has shown promising clinical responses. Of the 36 patients who received bintrafusp alfa, there was a clinical response rate of 38.9% with an acceptable safety profile (40). Prior studies with anti–PD-1/PD-L1 agents have demonstrated 15%–25% response rates in this patient population. Furthermore, patients who received bintrafusp alfa generated de novo HPV-specific T cell responses, and those patients with clinical responses induced higher levels of HPV-specific T cells (41). Since patients with HPV-associated malignancies have derived a clear benefit from treatment with bintrafusp alfa, one would conclude that the combination with a therapeutic HPV vaccine may prove beneficial.

In summary, this is the first study to our knowledge to report on a recombinant gorilla adenovirus vaccine for potential therapeutic use in HPV^+^ malignancies. These studies provide the rationale for potential clinical studies of PRGN-2009. A phase I study of PRGN-2009 alone, or in combination with bintrafusp alfa, is currently ongoing at the NCI, NIH (NCT04432597; https://clinicaltrials.gov/ct2/show/NCT04432597).

## Methods

### Experimental reagents.

The GC46 gorilla adenovector was identified and isolated from nonhuman primate sources (25, 26), and multiple genes (E1, E3, and E4) have been deleted to prevent viral replication (27). Five different HPV vaccine constructs were designed as part of the Cooperative Research and Development Agreement (CRADA) between the NCI, NIH, and Precigen Inc. Precigen Inc.’s UltraVector technology was used to optimize the vaccine design. After in vitro and in vivo evaluations, a construct designated PRGN-2009 was chosen for further studies. The PRGN-2009 vaccine design contains 35 non–HLA-restricted CTL epitopes of HPV 16 and 18 connected by linkers, including 3 HLA-A2 agonist epitopes of HPV16 previously identified by our laboratory (28). The vaccine or the GC46 empty vector control was given as an s.c. injection of 1 × 10^9^ VP once per week.

### Cell lines.

The TC-1 murine HPV16^+^ lung carcinoma cell line was a gift from T.C. Wu (Johns Hopkins University, Baltimore, Maryland, USA) and was cultured according to previous studies (42). TC-1 was derived from primary epithelial cells of C57BL/6 mice transfected with the HPV16 E6, HPV16 E7, and c-Ha-ras oncogenes (43). The SiHa human HPV^+^ squamous cell carcinoma cell line and the THP-1 DC line were obtained from ATCC and cultured according to the manufacturer’s specifications. Healthy-donor PBMCs were obtained from the NIH Blood Bank (NCT00001846), processed, and stored as previously described (44).

### Murine studies.

For syngeneic tumor studies, 2 × 10^4^ TC-1 cells were combined with Matrigel (Corning Life Sciences) at a 1:1 ratio immediately prior to s.c. injection into the right flank of C57BL/6 mice in a total volume of 100 μL. Four days after tumor injection, weekly treatments were started. For studies in the humanized mouse model, 2 × 10^6^ SiHa cells were combined with Matrigel at a 1:1 ratio immediately prior to s.c. injection into the right flank in a total volume of 100 μL. Seven days after tumor injection, PBMCs from a healthy donor were thawed in a 37°C water bath, followed by 3 washes with RPMI containing 10% FBS, and 1 wash with PBS. For PBMC reconstitution of the mice, 1 × 10^7^ PBMCs were injected i.p. in 200 μL PBS. Seven to 14 days after PBMC reconstitution (depending on tumor size), weekly treatments were started.

### In vivo CD4^+^ and CD8^+^ T cell depletion studies.

Mice were treated with CD4-depleting (clone GK1.5) or CD8-depleting (clone 2.43) antibodies (100 μg i.p., 3 doses, both from BioXCell) prior to TC-1 tumor instillation and then weekly after instillation; mice were bled to confirm depletion of T cell subsets by flow cytometry. When tumors reached approximately 70–100 mm^3^ in size, treatment with PRGN-2009 or empty vector control was started.

### In vitro evaluation of HPV vaccine constructs.

DCs were generated from human healthy-donor PBMCs and infected with the 5 HPV vaccine constructs at a multiplicity of infection (MOI) of 5000 VP per cell for 3 hours at 37°C. After 24 hours, autologous PBMCs were added to the culture at a 10:1 ratio for the first IVS. A second IVS cycle of these PBMCs was performed in the same manner with HPV vaccine–infected DCs at an MOI of 5000. IVS 3 was completed using the DC line THP-1 (ATCC), pulsed for 2 hours with HPV16 E6 or E7 15-mer peptides (0.0625 μg/μL) before adding the IVS 2 cells. IVS 3 was performed using THP-1 with IL-7 (10 ng/mL) and IL-15 (10 ng/mL) cytokine support. IVS 4 was completed in the same manner as IVS 3, and supernatants were collected 24 hours later for IFN-γ ELISA analysis (Human IFN-γ ELISA kit KHC4521, Invitrogen).

### Flow cytometry.

Tumors were excised and homogenized via mechanical dissociation, and single-cell suspensions were prepared by filtering through a 40 μm nylon cell strainer. Cell suspensions were stained on ice with fluorescently conjugated antibodies diluted in FACS buffer. Dead cells were identified via Live/Dead fixable stain (Thermo Fisher Scientific). Murine cells were stained with anti-mouse CD3 (clone 500A2), CD4 (clone RM4-5), CD8 (clone 53-6.7), CD45 (clone 30-F11), Ki67 (clone B56), CD44 (clone 1M7), CD62L (clone MEL-14), CD25 (clone PC61), F4/80 (clone BM8), GR1 (clone RB6-8C5), FoxP3 (clone FJk-16s), MHC-II (clone M5/114.15.2), CD11b (clone M1/70), Ly6C (clone AL-21), Ly6G (clone 1A8), CD206 (clone C068C2), PD-1 (clone J43), Tim-3 (clone B8.2C12), and CD38 (clone 90). Where indicated, intracellular staining was performed using the FoxP3/Transcription Factor kit (eBioscience), according to the manufacturer’s instructions. Cells were enumerated utilizing AccuCheck Counting Beads (Thermo Fisher Scientific). T cell IFN-γ production was detected via ex vivo incubation at 37°C with Cell Stimulation Cocktail (eBioscience) diluted 1:500 in RPMI for 5 hours with the addition of GolgiPlug (Thermo Fisher Scientific) for the final 4.5 hours of the culture, and intracellular staining for IFN-γ (clone XMG1.2) and GzmB (clone GB11). Antibodies used for flow cytometry were purchased from BioLegend. Cytometric data were obtained via a 4 laser Attune Flow Cytometer (Thermo Fisher Scientific). Data were analyzed with FlowJo (45).

### ELIspot assay.

Splenocytes from C57BL/6 mice were harvested, and ex vivo antigen-dependent cytokine secretion was assessed using an IFN-γ ELIspot kit, according to the manufacturer’s instructions (BD Biosciences). Overlapping 15-mer peptides (JPT Peptide Technologies Inc.) from HPV16 E6/E7, HPV18 E6/E7, and HIV-Gag (negative control) at a final concentration of 10 mg/mL were incubated with 2.5 × 10^5^ splenocytes per well in triplicate wells overnight. PMA stimulation was used as a positive control. ELIspot data are presented as the number of spot-forming cells (SFC) per 2.5 × 10^5^ splenocytes after subtracting the number of spots in paired wells containing control peptide (HIV-Gag) (46).

### Antigen-specific analyses of TILs.

At the end of study, CD45^+^ TILs were isolated using a mouse CD45^+^ isolation kit (Miltenyi Biotec) and stimulated overnight in triplicate wells with combined overlapping HPV16 E6/E7 15-mer peptides or DMSO (negative control). The cells were then stained for CD8, IFN-γ, and GzmB and evaluated by flow cytometry. Unstimulated TILs were also stained with CD8 and the mouse HPV16 E7 tetramer (RAHYNIVTF, catalog MHC-LC291, Creative Biolabs) and analyzed by flow cytometry. Cytometric data were obtained via a 4 laser Attune Flow Cytometer (Thermo Fisher Scientific). Data were analyzed with FlowJo (45).

### Histology.

IHC for CD8^+^ and CD4^+^ T cells was performed using the Perkin Elmer Opal IHC kit, according to the manufacturer’s instructions (Perkin Elmer) and imaged using an AxioScan Zeiss Imager.

### Statistics.

Statistical analyses were performed in GraphPad Prism 7 (GraphPad Software), using Mann-Whitney *U* and Kruskal-Wallis tests as appropriate. Repeated-measures 2-way ANOVA was used to analyze tumor growth curves. *P <* 0.05 was considered significant. Significance is indicated within figures as follows: *P < 0.05; **P < 0.01; ***P < 0.001; ****P < 0.0001.

### Study approval.

C57BL/6 mice were bred and maintained in microisolator cages under SPF conditions in accordance with the Association for Assessment and Accreditation of Laboratory Animal Care (AAALAC) guidelines at the NIH. NSG-β2m^–/–^ mice were obtained from the Jackson Laboratory, and they were bred and maintained in accordance with AAALAC guidelines. All animal studies were performed following approval from the NIH IACUC (protocol no. LTIB-057).

## Author contributions

STP, JS, DEB, HS, and CJ designed the research studies. STP, CSR, YMM, DEB, LKP, and NR conducted the experiments. STP, CSR, YMM, DEB, LKP, and NR acquired the data. CJ, JS, DEB, HS, STP, LKP, and CSR analyzed and interpreted the data. STP, JS, and CJ wrote the manuscript. All authors read and approved the final manuscript. STP and CSR are co–first authors; order of authorship was chosen based on the fact that STP was the primary responsible scientist for the in vivo studies.

## Supplementary Material

Supplemental data

## Figures and Tables

**Table 1:**
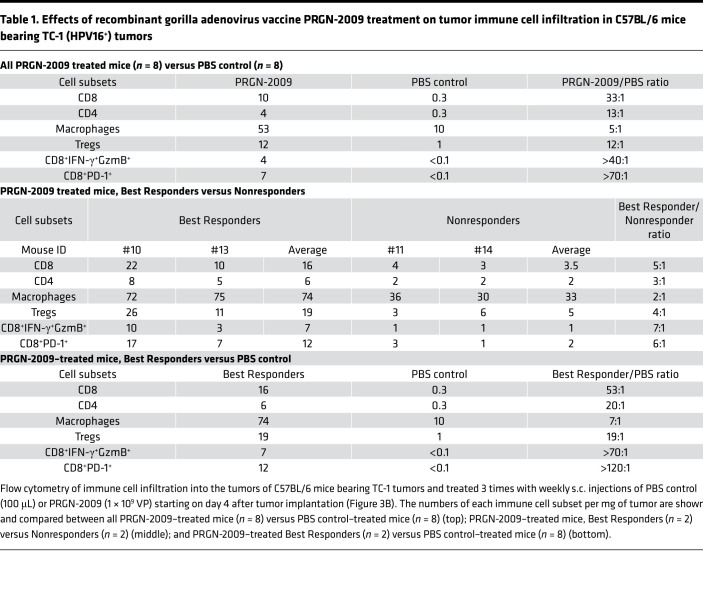
Effects of recombinant gorilla adenovirus vaccine PRGN-2009 treatment on tumor immune cell infiltration in C57BL/6 mice bearing TC-1 (HPV16^+^) tumors

## Version 1 03/02/2021

In-Press Preview

## Version 2 04/08/2021

Electronic publication
