# Supplementary material for: Characterization of recombinant gorilla adenovirus HPV therapeutic vaccine PRGN-2009
Source: JCI Insight. 2021 Apr 8;6(7):e141912. doi: 10.1172/jci.insight.141912 (PMC8119209; doi:10.1172/jci.insight.141912)
Supplement: Supplemental data [file jciinsight-6-141912-s076.pdf]

Supplemental Table 1: Effects of recombinant gorilla adenovirus vaccine PRGN-2009 treatment on tumor immune cell infiltration in C57BL/6 mice bearing TC-1 ($HPV16^{+}$) tumors

All PRGN-2009 treated mice (n=8) vs. PBS control (n=8)

Cell Subsets	PRGN-2009	PBS Control	PRGN-2009:PBS Ratio
NK	4	2	2 : 1
MDSC	550	257	2 : 1
$CD8^{+}IFN\gamma^{+}$	2	< 0.1	> 20 : 1
$CD8^{+}GzmB^{+}$	2	< 0.1	> 20 : 1
$CD4^{+}IFN\gamma^{+}$	1.5	< 0.1	> 15 : 1
$CD4^{+}PD\text{-}1^{+}$	2	< 0.1	> 20 : 1

PRGN-2009 treated mice, Best Responders vs. Non-Responders

Cell Subsets	Best Responders			Non-Responders			Best:Non-Resp Ratio
Mouse ID	#10	#13	Avg	#11	#14	Avg	
NK	4	6	5	4	3	3.5	1.4 : 1
MDSC	188	377	283	859	776	818	0.3 : 1
$CD8^{+}IFN\gamma^{+}$	6	2	4	1	< 0.1	0.5	8 : 1
$CD8^{+}GzmB^{+}$	2	2	2	1	1	1	2 : 1
$CD4^{+}IFN\gamma^{+}$	3	2	2.5	1	< 0.1	0.5	5 : 1
$CD4^{+}PD\text{-}1^{+}$	5	3	4	2	< 0.1	1	4 : 1

PRGN-2009 treated mice, Best Responders vs. PBS Control

Cell Subsets	Best Responders	PBS Control	Best Resp:PBS Ratio
NK	5	2	3 : 1
MDSC	283	257	1.1 : 1
$CD8^{+}IFN\gamma^{+}$	4	< 0.1	> 40 : 1
$CD8^{+}GzmB^{+}$	2	< 0.1	> 20 : 1
$CD4^{+}IFN\gamma^{+}$	2.5	< 0.1	> 25 : 1
$CD4^{+}PD\text{-}1^{+}$	4	< 0.1	> 40 : 1

Flow cytometry of immune cell infiltration into the tumors of C57BL/6 mice bearing TC-1 tumors and treated three times with weekly s.c. injections of PBS control (100µl) or PRGN-2009 ($1x10^9$ VP) starting on day 4 post-tumor implantation. The numbers of each immune cell subset per mg of tumor are shown and compared between (top) All PRGN-2009 treated mice (n=8) vs. PBS control treated mice (n=8); (middle) PRGN-2009 treated mice, Best Responders (n=2) vs. Non-Responders (n=2); and (bottom) PRGN-2009 treated Best Responders (n=2) vs. PBS control treated mice (n=8).

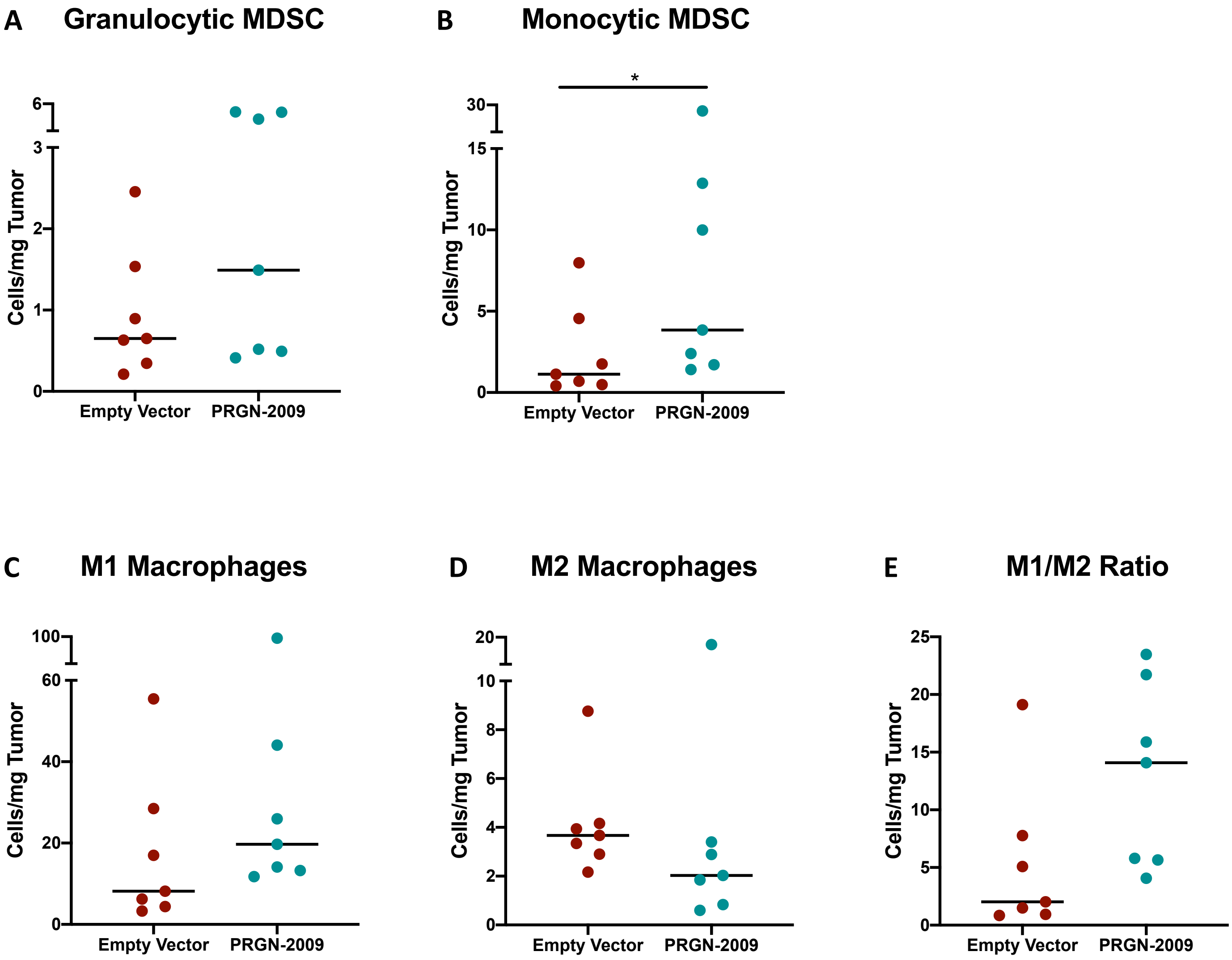

Supplemental Figure 1. MDSC and Macrophage populations in mice treated with PRGN-2009. C57BL/6 mice (n=7 per group) bearing s.c. TC-1 HPV16^{+} tumors were treated with empty vector control (1x10^{9} VP, s.c.) or PRGN-2009 (1x10^{9} VP, s.c.) on days 7 and 14 post-tumor implantation. Flow cytometry was performed on single cell suspensions of tumor tissue at the end of study (day 23). (**A**) Granulocytic MDSCs were defined as CD11b^{+}Ly6G^{+}. (**B**) Monocytic MDSCs were defined as CD11b^{+}Ly6C^{+}. (**C**) M1 macrophages were defined as CD11b^{+}F4/80^{+}MHC-II^{+}. (**D**) M2 macrophages were defined as CD11b^{+}F4/80^{+}CD206^{+}. (**E**) M1/M2 macrophage ratio. All myeloid subsets are shown per mg tumor. Mann-Whitney test. *$P < 0.05$.

MDSCs, myeloid-derived suppressor cells; s.c., subcutaneous.

A. Empty Vector B. PRGN-2009

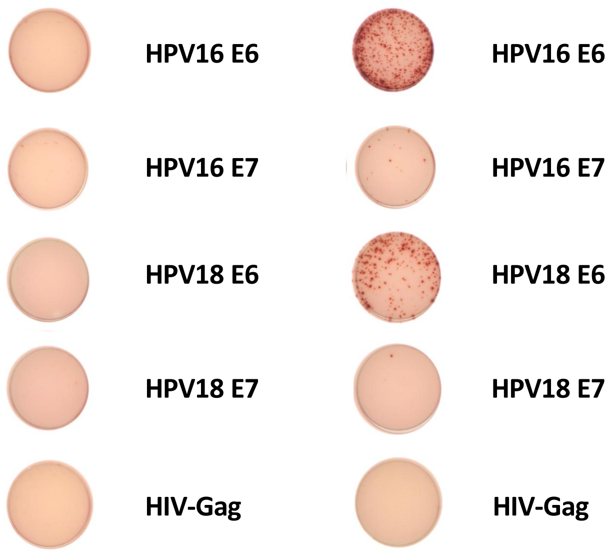

C.

	IFNγ Spots	
	Empty Vector	PRGN-2009
HPV16 E6	0	302
HPV16 E7	1	1
HPV18 E6	1	43
HPV18 E7	1	2
HIV-Gag	0	0

Supplemental Figure 2. HPV-specific T cells in the periphery induced by PRGN-2009. C57BL/6 mice (n= 6-7 per group) bearing s.c. TC-1 HPV16^{+} murine tumors were treated with empty vector ($1x10^9$ VP, s.c.) or PRGN-2009 ($1x10^9$ VP, s.c.) on days 7 and 14 post-tumor implantation. At the end of study (day 23), an ELIspot assay was performed on splenocytes using overlapping 15-mer peptides for HPV16 E6, HPV16 E7, HPV18 E6, and HPV18 E7. Representative photomicrographs of IFNγ spots are shown for splenocytes from mice treated with (**A**) empty vector, and (**B**) PRGN-2009, after stimulation with the indicated 15-mer peptides. The median number of spot forming cells per well are shown in (**C**).
s.c., subcutaneous; VP, virus particles.

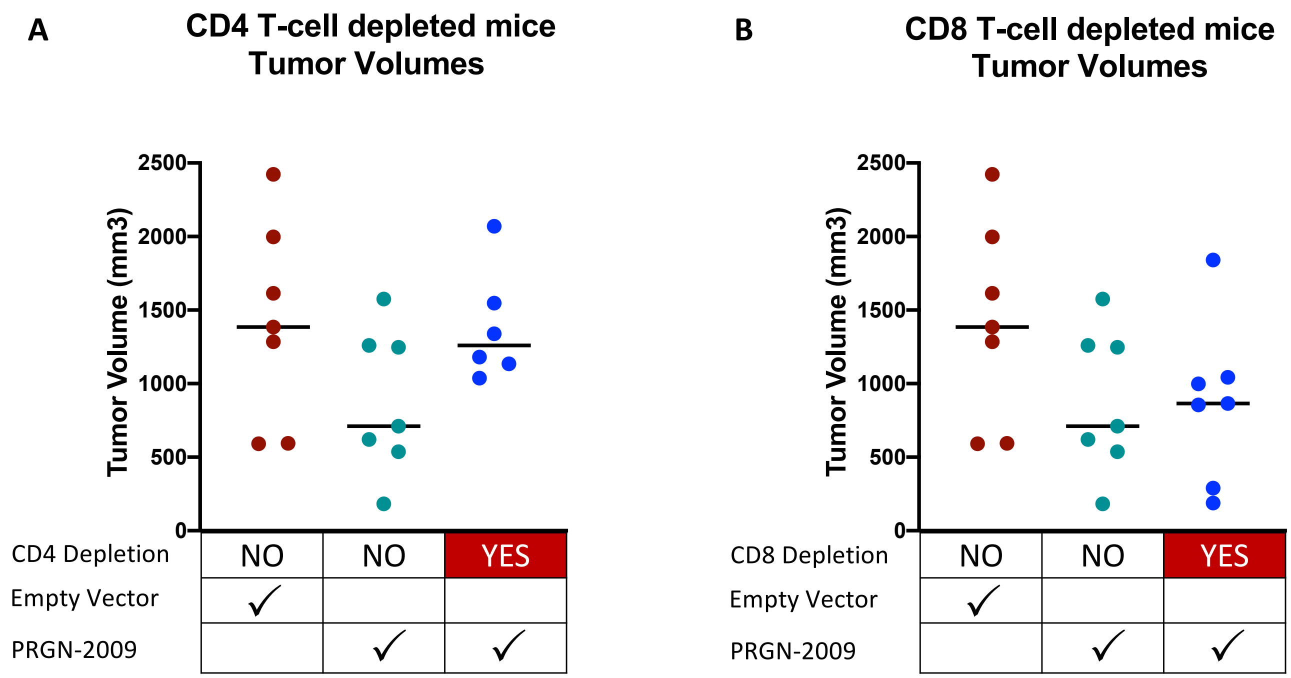

C

T-cell depleted mice
Tumor Volumes

# Mice with tumor volume < 800 mm^3	
Empty Vector	**2 / 7 (29%)**
PRGN-2009	**4 / 7 (57%)**
CD4 Depleted + PRGN-2009	**0 / 6 (0%)**
CD8 Depleted + PRGN-2009	**2 / 7 (29%)**

Supplemental Figure 3. CD4 and CD8 T-cell contribution to the anti-tumor effects of PRGN-2009. C57BL/6 mice (n= 6-7 per group) were depleted of CD4 or CD8 T-cell populations using commercially obtained depleting antibodies (Methods). Mice were instilled with $2x10^4$ TC-1 $HPV16^+$ murine cancer cells s.c., and treated with weekly injections of empty vector control ($1x10^9$ VP, s.c.) or PRGN-2009 ($1x10^9$ VP, s.c.) starting on day 7 post-tumor implantation. Treatments were administered on days 7 and 14. The final tumor volumes (day 22) are shown for (**A**) CD4 depleted mice treated with PRGN-2009 and (**B**) CD8 depleted mice treated with PRGN-2009. (**C**) Table showing the number of mice in each group with a tumor volume below 800 mm^3. s.c., subcutaneous.

Supplemental Table 2: Safety and tolerability of repeat subcutaneous administration of recombinant gorilla adenovirus vaccine PRGN-2009

Body Weights

	Body Weights (g)				
	**Day -8**	**Day 0**	**Day 7**	**Day 14**	**Day 21**
PRGN-2009 Mean (SD)	15.07 (1.49)	15.43 (1.53)	16.00 (1.45)	16.47 (1.57)	17.18 (1.42)
Empty Vector Mean (SD)	14.92 (0.80)	15.85 (0.52)	16.62 (0.61)	17.03 (0.69)	17.93 (0.65)
FFB Control Mean (SD)	14.96 (0.86)	16.23 (0.94)	17.17 (0.94)	17.80 (0.71)	18.82 (0.97)

Organ Weights

	Organ Weights (mg)					
	**Duodenum**	**Heart**	**Kidneys**	**Liver**	**Lungs**	**Brain**
PRGN-2009 Mean (SD)	114.0 (35.1)	97.2 (8.4)	232.8 (14.3)	794.0 (70.7)	438.7 (43.3)	414.7 (55.8)
Empty Vector Mean (SD)	113.5 (27.2)	102.5 (20.5)	225.3 (19.3)	812.5 (140.0)	415.3 (89.3)	442.2 (28.5)
FFB Control Mean (SD)	107.3 (18.3)	126.3 (64.0)	211.7 (49.1)	820.3 (129.1)	367.2 (61.0)	452.5 (25.0)

C57BL/6 mice (n=6 per group) were treated three times with weekly injections of PRGN-2009 ($1x10^{10}$ VP), empty vector control ($1x10^{10}$ VP), or FFB control (100µl), s.c. All mice were monitored for any clinical signs and weighed during the course of the study (top). Average body weights (SD) per group are shown at randomization (day -8) and weekly time points. On day 21, mice were sacrificed and examined for gross pathological changes. The liver, duodenum, kidney, brain, heart and lungs were harvested and weighed (bottom). The average organ weights (SD) per group are shown. No significant differences were observed between the treatment groups. FFB, final formulation buffer; VP, virus particles; SD, standard deviation
